# Local Electrochemical Corrosion of 6061 Aluminum Alloy with Nano-SiO_2_/MAO Composite Coating

**DOI:** 10.3390/ma16206721

**Published:** 2023-10-17

**Authors:** Jie Zhao, Hongwei Zhang, Xiaoyu Yang, Yanhong Gu, Yida Liu

**Affiliations:** 1 School of Safety Engineering, Beijing Institute of Petrochemical Technology, Beijing 102617, China; 2 School of Mechanical Engineering, Beijing Institute of Petrochemical Technology, Beijing 102617, China

**Keywords:** 6061 aluminum alloy, micro-arc oxidation, nano-SiO_2_, composite coating, local electrochemistry

## Abstract

To improve the corrosion resistance of 6061 Al in electric vehicle battery packs, a composite coating of nano-SiO_2_/Micro-Arc oxidation (MAO) ceramic structure was prepared on its surface. Electrochemical impedance spectroscopy (EIS) and potentiodynamic polarization curves (PDP) were used to evaluate the corrosion resistance of the specimens after 7 days immersion in a 3.5% NaCl solution. The corrosion resistance of the prefabricated coatings was measured via local electrochemical impedance spectroscopy (LEIS). Confocal microscopy, scanning electron microscopy (SEM), and X-ray diffraction (XRD) were used to characterize the microstructure and phase composition of the specimens. An energy dispersive spectrometer (EDS) was used to detect the elemental composition of the surface of the specimen. The results showed that the specimen with nano-SiO_2_/MAO composite coating had the least amount of micropores and superior corrosion resistance. The global electrochemical impedance of nano-SiO_2_/MAO composite coating was 1.1 times higher than that of the MAO coating and 8.4 times higher than that of the 6061 Al. When the coating was defective, the local electrochemical impedance of the nano-SiO_2_/MAO composite coating was still two times higher than that of the MAO coating. In the presence of scratches, the nano-SiO_2_/MAO composite coating still showed high corrosion resistance. The collapse corrosion mechanism of the nano-SiO_2_/MAO composite coating was proposed.

## 1. Introduction

In the field of new energy vehicles, aluminum alloy is usually used to make battery packs to reduce the weight of the vehicle. Meanwhile, in order to avoid affecting the heat dissipation of the battery, aluminum alloy battery packs are also part of the vehicle chassis [1]. However, the corrosion resistance of the aluminum alloy is poor, especially when used in the lightweight design of automobile chassis, which can be damaged by scratches, rain and snow, and snow melting agent. Therefore, it is important to enhance the corrosion resistance of aluminum alloy surfaces. Recently, aluminum alloy coating preparation methods include anodic oxidation [2], physical vapor deposition [3], electrochemical coating [4], and organic coating [5]. In recent years, the micro-arc oxidation technology developed on the basis of anodic oxidation has received wide attention [6]. Micro-arc oxidation is characterized by low preparation cost, easy realization, and better coating performance.

The micro-arc oxidation (MAO) process is commonly used to enhance the surface properties of aluminum alloy [7,8,9], form nano-ceramic coatings consisting of γ-Al_2_O_3_ and α-Al_2_O_3_ phases on aluminum alloys [10,11,12], and enhance the corrosion resistance of aluminum alloys [13,14,15,16,17]. However, many micropores are produced during micro-arc oxidation [18,19], which are easily corroded by the corrosive fluid. Some micropores can be eliminated by adding nano-particles [20]. Some scholars [21,22] added nano-SiO_2_ particles to the aluminum alloy during micro-arc oxidation, which improved the thickness and compactness of the coating and reduced the porosity of the coating, and effectively improved the surface properties of the coating. Yang et al. [23] and Nadimi et al. [24] prepared micro-arc oxidation coatings with the addition of SiO_2_ nanoparticles on the surface of magnesium and aluminum alloys, respectively, and the experimental results showed that the addition of SiO_2_ nanoparticles enhanced the corrosion resistance of the coatings. However, in practical application, the aluminum alloy battery pack of the automobile will be damaged by scratches due to the complex road environment, which will affect the local corrosion resistance. Therefore, it is essential to study the local impedance of micro-arc oxidation coating damaged by scratches. The local electrochemical impedance spectrum (LEIS) technique is one of the most widely used analytical techniques for micron-sized MAO coatings, which can accurately and intuitively analyze the impedance of defects in the coatings [25,26]. Ma et al. [27] and Dai et al. [28] used LEIS technology to evaluate the local corrosion resistance of the MAO coating scratches on magnesium alloys, and accurately analyzed the mechanism of local micro-pore corrosion. Therefore, it is very significant to study the corrosion resistance of aluminum alloy coating damaged by scratches via LEIS technology.

In this paper, MAO and nano-SiO_2_/MAO composite coatings were prepared on the surface of 6061 Al, and their corrosion resistance was evaluated in a 3.5% NaCl solution (simulated snowmelt) at 55 °C (upper-temperature limit of the battery pack). Confocal microscope, scanning electron microscope (SEM), X-ray diffraction (XRD), and energy dispersion spectrometer (EDS) were used to observe the microstructure, phase composition, and element composition of the coating before and after corrosion. The local corrosion law of the coating before and after scratching was studied via LEIS, and the corrosion mechanism was proposed.

## 2. Materials and Methods

### 2.1. Specimens Preparation

The aluminum alloy substrate used in the experiment is 6061-T6 aluminum alloy produced by Mingtai Industry Co., LTD. from Zhengzhou, China, and its chemical composition (wt%) is 1.025 Mg, 0.636 Si, 0.243 Cu, 0.125 Mn, 0.025 Ti, 0.109 Cr, 0.506 Fe, 0.151 Zn, and the balance of Al. The aluminum alloy specimens with dimensions of 30 × 40 × 5 mm were cut into blocks with a cross-sectional area of 1 cm^2^ and used for electrochemical experiment. Each experiment consists of three groups of parallel specimens. The specimens were sanded with sandpaper of 240#, 800#, and 1200#, and then cleaned with ultra-pure water. Finally, the specimens were immersed in a beaker filled with anhydrous ethanol for liquid sealing.

According to the size of the electrolytic cell and the specimen, 3000 mL micro-arc oxidation electrolyte should be prepared, including NaOH 6 g, Na_2_SiO_3_ 30 g, (NaPO_3_)_6_ 45 g, and nano-SiO_2_ powder 9 g. Chemical reagents are prepared by Hengxing Chemical Preparation Co., LTD. from Tianjin, China. The above solution was divided into two kinds: one was the ordinary micro-arc oxidation electrolyte, excluding nano-SiO_2_ powder, and the other was a composite micro-arc oxidation electrolyte, containing nano-SiO_2_ powder.

**Figure 1:**
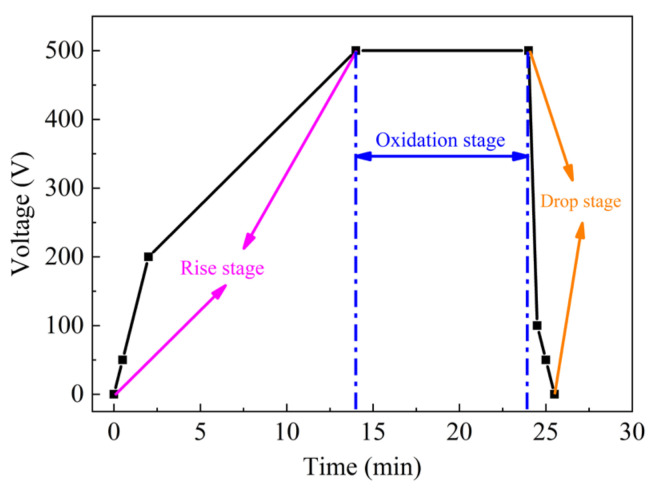
Micro-arc oxidation voltage curve.

The equipment used in the MAO process was manufactured by Haoning™ Electronic Technology Co., LTD. from Xi’an, China (Model HNMAO-20). The coating preparation process used the constant voltage boosted method (Figure 1). The initial voltage was 50 V, and the ramp-up rate was 50 V every 30 s until 200 V. The voltage was increased from 200 to 500 V at a rate of 25 V/min, after which the micro-arc oxidation was maintained for 10 min. The voltage drop phase was completed in less than a minute, and the steps were reduced from 500 V to 100 V, then to 50 V, and finally to 0 V.

Micro-arc oxidation is divided into four stages: The first stage (Rise phase) is the anodic oxidation stage, in which the applied voltage is gradually increased, and the oxidation film of small thickness is formed on the surface of the substrate. The second stage (Oxidation phase) is the breakdown discharge stage, in which the voltage continues to increase, the breakdown occurs in the weak position of the oxide film, the generated high current makes the substrate surface to form a plasma discharge channel, and spark discharge phenomenon occurs. The third stage (Oxidation phase) is the coating layer thickening stage, in which after the oxidation film is broken down, the energy released leads to the melting of the oxide, metal matrix, etc., which is rapidly cooled after contacting the electrolyte, and the generated oxide gathers around the discharge channel, and the thickness of the coating layer is uniformly increased. The fourth stage (Drop phase) is the micro-arc oxidation stop stage, in which when the coating thickness increases to the point where the applied voltage cannot break down the micro-arc oxidized coating layer, the micro-arc oxidation process stops.

### 2.2. Surface Characteristics

The surface morphology of the coating was observed via the SEM (SSX-550, SHIMADZU from Tsushima, Japan). The elements on the surface of coating were detected via the EDS (AZtecTEM, Oxford Instruments from Oxford, UK) surface scanning method, and the elements in the thickness direction of the coating section were detected via the EDS line scanning method. XRD (D8-FOCUS, BRUKER from Brook, Germany) using a Cu Kα radiation was used to detect the phase composition of the coating before and after corrosion. The grazing angle was 2°, the scanning range was 10~90°, and the scanning speed was 5°/min. The corrosion of the damaged coating was observed using a confocal microscope (KH-8700, HIROX from Shanghai, China). LEIS (AMETEK, VersaSCAN^TM^, Princeton, NJ, USA)was used to test the three-dimensional micropore distribution and local electrochemical behavior with a test area of 2 × 2 mm^2^.

### 2.3. Electrochemical Experiments

#### 2.3.1. Global Electrochemical Measurement

The working station (AMETEK, VersaSTAT-3F, Princeton, NJ, USA) was used for the global electrochemical test. The traditional three-electrode system was adopted. The global electrochemical test included electrochemical impedance spectroscopy (EIS) and potentiodynamic polarization (PDP). The corrosion solution was 3.5% NaCl solution.

The frequency scanning range of electrochemical impedance spectroscopy was 10^−2^~10^5^ Hz, and the excitation voltage was 10 mV. The voltage scanning range of the potentiodynamic polarization test was −250~500 mV, step height was 1 mV, step time was 2 s, and scanning rate was 0.5 mV/s.

#### 2.3.2. Local Electrochemical Measurement

The Local electrochemical tests were measured using a VersaSCAN^TM^ (Princeton, NJ, USA) electrochemical scanning system. A four-electrode system was used as a corrosion cell with the specimen as the working electrode, saturated calomel as the reference electrode, a platinum plate below the probe (P/N-224114) as the counter electrode, and the probe as the measuring electrode with a probe radius of 10 μm. During the local electrochemical test, the excitation voltage was 10 mV, the tip of the probe was 100 μm from the specimen surface, the test frequency was fixed at 1000 Hz, and the conductivity of the electrolyte was 5.6 S/m.

### 2.4. Numerical Simulation Scheme for Long-Period Corrosion Prediction

#### 2.4.1. Models and Grids

**Figure 2:**
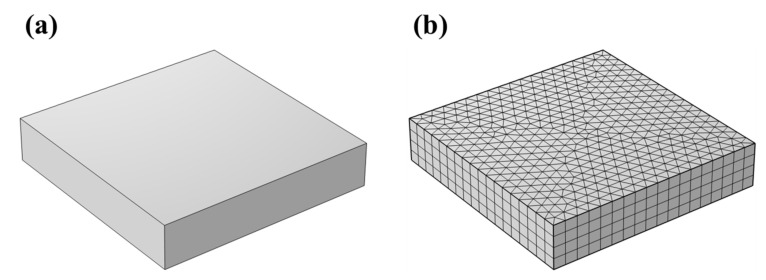
Long-period corrosion simulation in 3D: (**a**) geometric modeling; (**b**) meshing.

The COMSOL Multiphysics 5.6 finite element analysis software was used to establish a rectangular body with the size of 10 × 10 × 2 mm as a model, and the mesh was divided via the method of “Structured free triangle mesh + Face sweep”, as shown in Figure 2.

#### 2.4.2. Control Equations and Boundary Conditions

Numerical simulations make the following assumptions:

(1) The corrosion electrolyte solution is well mixed and the concentration gradient is not considered. (2) The corrosion electrolyte solution is electrically neutral and convective diffusion effects are not considered. (3) The corrosion electrolyte solution is incompressible. (4) The electrolyte domain has no specific size, and all outside the electrode domain are set as infinite electrolyte domain. (5) The electrode domain is isotropic. (6) The kinetic process of the electrode reaction is rapid, with negligible activation loss due to charge transfer. (7) Substance dissolution occurs at the electrode surface.

The corrosion process is a charge transfer between the corrosion solution and the electrode surface, where the current–voltage relationship follows Ohm’s law and the current conservation equation. Both Na^+^ and Cl^−^ in the corrosive solution are ionic conductors and therefore also follow Faraday’s law, and their net current density can be described by the sum of the fluxes of all ions as Equation (1):

(1) $$i_{l}=F\underset{i}{\sum }z_{i}N_{i}$$

where $i_{l}$ is the electrolyte current density, *F* is the Faraday constant, $z_{i}$ is the charge of ion *i*, and $N_{i}$ is the flux of ion *i*. The flux of a single ion can be described by the Nernst–Planck equation as Equation (2):

(2) $$N_{i}=-D_{i}\nabla c_{i}-z_{i}Fu_{i}c_{i}\nabla \Phi_{l}+c_{i}V$$

where $D_{i}$ is the diffusion coefficient of ion *i*, $c_{i}$ is the concentration of ion *i*, $u_{i}$ is the mobility of ion *i*, $\Phi_{l}$ is the electrolyte potential, and *V* is the convection rate. According to assumption 1 and assumption 2, the convection and diffusion terms in Equation (2) are both zero; therefore, the simplified Nernst–Planck equation can be obtained as Equation (3):

(3) $$N_{i}=-z_{i}Fu_{i}c_{i}\nabla \Phi_{l}$$

The current conservation equation containing a general current source term is based on the assumption of electroneutrality as Equation (4):

(4) $$\nabla \cdot i_{l}=0$$

Based on the above analysis, the control equation is obtained as Equation (5):

(5) $$\left\{\begin{array}{c} i_{l}=-F^{2}\sum z_{i}^{2}u_{i}c_{i}\nabla \Phi_{l}\\ \nabla \cdot i_{l}=0 \end{array}\right.$$

The electrode polarization in the corrosion process can be non-negligible, and the long-period corrosion prediction needs to use the kinetic potential polarization curve experimental data as boundary conditions; therefore, the net polarization current density at the anode when dissolution occurs and the polarization potential of the whole corrosion system conforms to the Butler–Volmer electrode kinetic equation as Equation (6):

(6) $$\left\{\begin{array}{c} i_{loc}=i_{corr}\left[exp\left(\frac{\alpha_{a}F\eta }{RT}\right)-exp\left(\frac{\alpha_{c}F\eta }{RT}\right)\right]\\ \eta =\phi_{s,ext}-\phi_{l}-E_{eq} \end{array}\right.$$

where $i_{loc}$ is the net polarization current density of the electrode, $i_{corr}$ is the corrosion current density in the measured PDP curve, $\alpha_{a}$ is the anodic transfer coefficient, $\alpha_{c}$ is the cathodic transfer coefficient, *F* is the Faraday constant, *η* is the overpotential, *R* is the gas constant, *T* is the absolute temperature, $\phi_{s,ext}$ is the external potential.,$\phi_{l}$ is the electrolyte potential, and $E_{eq}$ is the corrosion potential in the measured PDP curve.

## 3. Results and Discussion

### 3.1. Characterization of MAO Coatings

#### 3.1.1. Surface and Cross-Section Morphology

**Figure 3:**
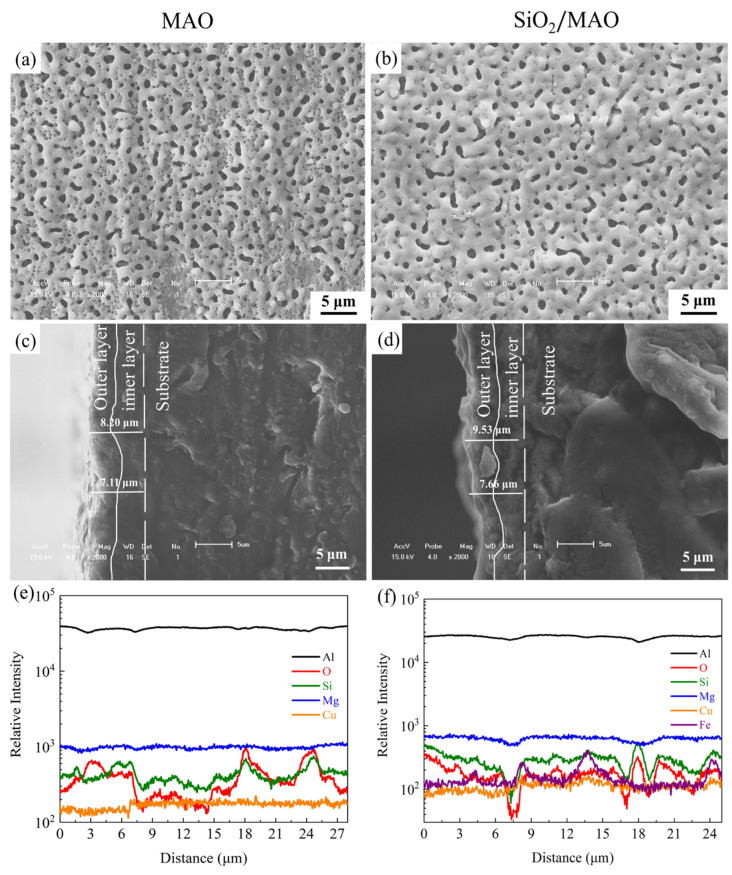
Surface morphology and cross-section morphology and elemental analysis results: (**a**,**c**,**e**) the MAO coating; (**b**,**d**,**f**) nano-SiO_2_/MAO composite coating.

Figure 3 shows the SEM morphology of the MAO coating and the nano-SiO_2_/MAO composite coating, and the cross-section elements of the two coatings. By comparing the morphology of micropores of the two coatings, there are many micropores on the surface of the MAO coating in Figure 3a, while there are few micropores on the surface of the nano-SiO_2_/MAO composite coating in Figure 3b, and most of the micropores have disappeared, indicating that the addition of nano-SiO_2_ particles contributed to eliminate some micropores. The main component of micro-arc oxidation coating grown in situ on the surface of aluminum alloy substrate is Al_2_O_3_ [29]. Therefore, the two most surface elements detected are Al and O in Figure 3a,b. Mg comes from 6061 Al itself, and P comes from the electrolyte of the micro-arc oxidation process. Si element comes from 6061 Al itself and the electrolyte of the micro-arc oxidation process.

Figure 3c–f show the cross-section thickness of the two micro-arc oxidation coatings and EDS line scanning results of elements. The MAO coating grown in situ adheres to the substrate to a better extent, and the boundary between the metal and the coating is not obvious [7]. As shown in Figure 3c,d, the thickness of MAO coating is 7.1–8.2 μm, and that of nano-SiO_2_/MAO composite coating is 7.7–9.6 μm. The place where the elemental content of Si and O decreases is the boundary between the coating and the substrate. There are also Mg elements in both the coating and the substrate, while Cu element and Fe element only present in the substrate. Therefore, the Cu element increases slightly along the scan line. The Mg elements content is unchanged.

**Figure 4:**
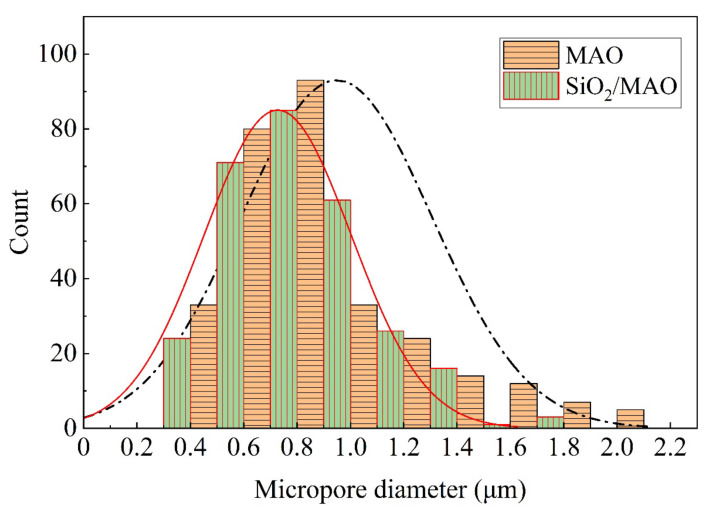
Distribution of microporous diameter on MAO coating and nano-SiO_2_/MAO composite coating.

Figure 4 shows the micropore diameters distribution measured by three parallel experiments for the MAO coating and the nano-SiO_2_/MAO composite coating. The micropore size of the MAO coating ranges from 0.4 μm to 2.1 μm, with an average diameter of about 0.9 μm. The micropore size of nano-SiO_2_/MAO composite coating ranges from 0.3 μm to 1.8 μm, with an average diameter of 0.7 μm. The main reason is that nano-SiO_2_ particles penetrate into the micropores of MAO coating, eliminating some of the micropores and making the coating more dense and complete.

#### 3.1.2. Three-Dimensional Micropore Distribution

**Figure 5:**
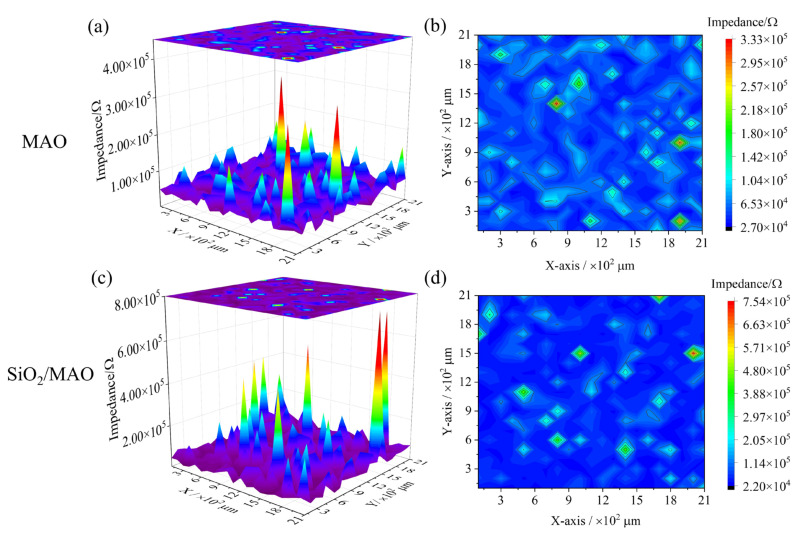
Three-dimensional micropore distribution of scratch-free coating: (**a**,**b**) MAO coating; (**c**,**d**) nano-SiO_2_/MAO composite coating.

Figure 5 shows the three-dimensional micropore distribution of the MAO coating and nano-SiO_2_/MAO composite coating without scratches at the same test frequency for an area of 2 × 2 mm^2^. The height and distribution of impedance peaks show the three-dimensional micropore distribution of the coating. The impedance of nano-SiO_2_/MAO composite coating (7.54 × 10^5^ Ω) is greater than that of the MAO coating (3.33 × 10^5^ Ω), and the overall impedance was more than doubled. Compared to the local impedance distribution of the MAO coating and nano-SiO_2_/MAO composite coating, the nano-SiO_2_/MAO composite coating has fewer convex peaks and more uniform distribution of micropores. This indicates that the addition nano-SiO_2_ can eliminate some micropores in the MAO coating and effectively improve the integrity of the coating.

#### 3.1.3. Phase Analysis

**Figure 6:**
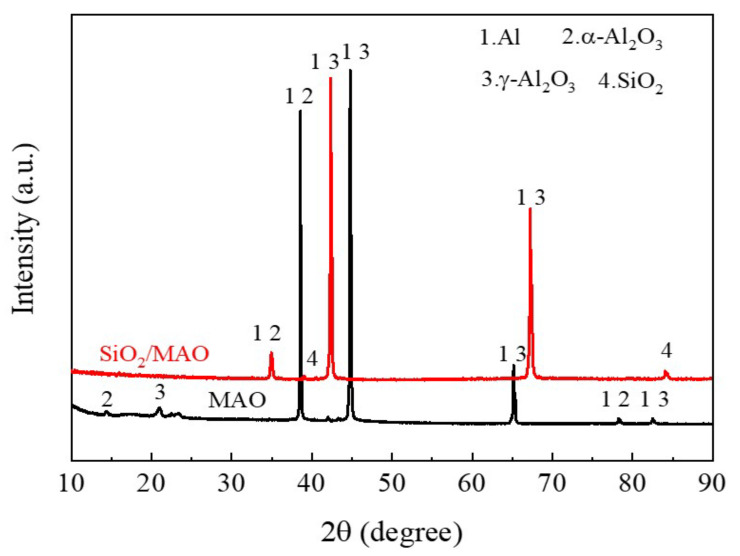
XRD scan results of MAO coating and nano-SiO_2_/MAO composite coating.

Figure 6 shows that the surface composition of the coating is detected using the X-ray diffractometer. The main peaks of the MAO coating and nano-SiO_2_/MAO composite coating were Al and Al_2_O_3_. The X-ray penetrated the coating and reached the aluminum alloy substrate, so Al was detected. Al_2_O_3_ is the main component of micro-arc oxidation coating, which can be divided into α crystal and γ crystal [10,14,17]. α-Al_2_O_3_ is a stable phase and γ-Al_2_O_3_ is a sub-stable phase, the nucleation rate of α-Al_2_O_3_ is lower than that of γ-Al_2_O_3_ at high cooling, and γ-Al_2_O_3_ is easily formed in the outer layer of the specimen at high cooling [29,30]. However, α-Al_2_O_3_ is easily formed in the inner holes and other film layers due to the small contact area with electrolyte and low thermal conductivity [14]. Therefore, the number of γ-Al_2_O_3_ peaks in the figure is in the majority. The peak of SiO_2_ indicates that the nano-particles have successfully entered the coating.

### 3.2. Global Electrochemical Behavior

#### 3.2.1. Electrochemical Analysis

**Figure 7:**
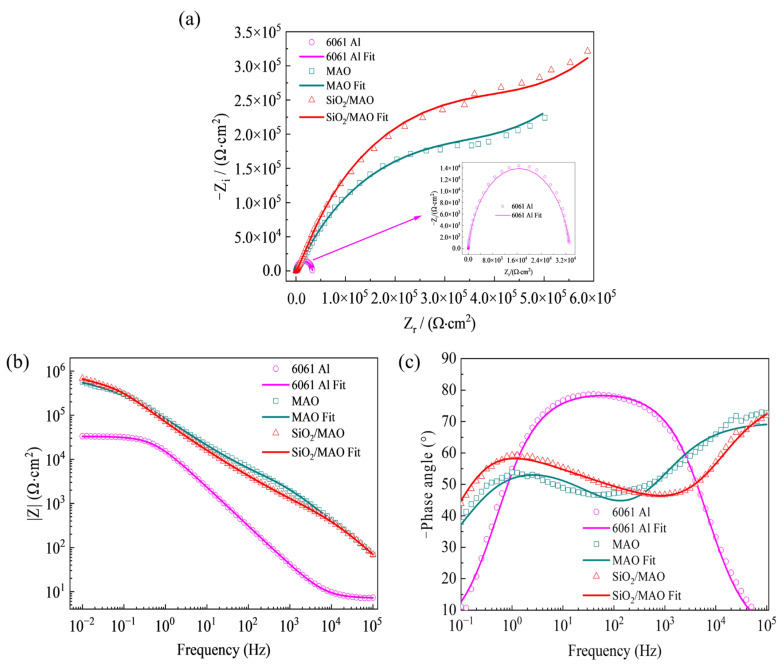
The Nyquist and Bode plots of the three specimens after immersion in 3.5% NaCl solution for 7 days: (**a**) Nyquist plot; (**b**) Bode impedance plot; (**c**) Bode phase plot.

The electrochemical impedance Nyquist curves of the three specimens are shown in Figure 7a. The measured values are represented by symbols, the solid line is the fitted value drawn according to the selected circuit, and the curve shape is approximately an arc. In the high-frequency region, the corrosion of 6061Al is mainly controlled by charge transfer, while in the low-frequency region, 6061Al corrosion is mainly controlled by substance transfer [9]. An approximately linear curve in the low-frequency area shows the existence of Warburg impedance (W component) [31]. The larger the radius of the impedance arc, the smaller the corrosion rate, and the better the corrosion resistance of the specimen. The impedance arc radius of the nano-SiO_2_/MAO composite coatings was much larger than that of the other specimens for 7 days of corrosion time, which indicates that the incorporation of nano-SiO_2_ enhances the corrosion resistance of the MAO coatings, and improves the protective performance of the coatings on 6061Al. In addition, pitting is the most common type of localized corrosion of aluminum alloy, especially in the solution containing Cl^−^ the oxide film on the surface of the metal substrate is easy to be corroded by the highly penetrating Cl^−^, accelerating the localized dissolution of the metal substrate and the occurrence of pitting. This is especially evident in coated specimens.

The electrochemical impedance Bode curves of the three specimens are shown in Figure 7b. The measured values are represented by symbols, and the solid line is the fitted value drawn according to the selected circuit. The impedance of the nano-SiO_2_/MAO composite coatings were significantly greater than that of the other specimens at corrosion times of 7 days. Compared with the 6061Al substrate, the coated specimens have two arcs in the mid- and high-frequency regions (Figure 7c), corresponding to the inner and outer layers. Comparison reveals that the arc of the nano-SiO_2_ composite coating is shifted towards higher frequencies, which further improves the corrosion resistance and stability of the nano-SiO_2_ composite coating. In addition, the incorporation of nano-SiO_2_ reduced the porosity of the MAO coating and inhibited the corrosion process.

**Figure 8:**
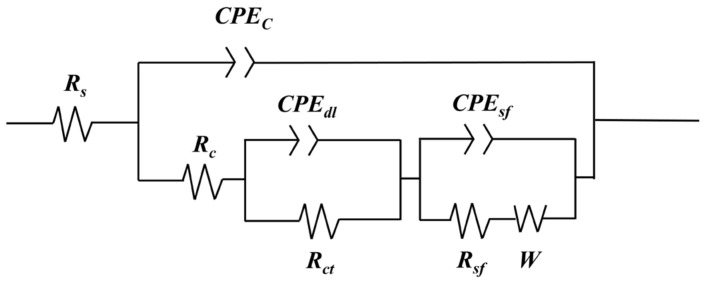
The equivalent electrical circuit of nano-SiO_2_/MAO composite coating after corrosion.

Figure 8 shows the equivalent circuit of nano-SiO_2_/MAO composite coating after corrosion in 3.5% NaCl solution. The coating was porous on the outside and dense on the inside. In the high-frequency region, which is mainly controlled by charge transfer, and in the low-frequency region, which is mainly controlled by matter transfer, a section of approximately linear curve appears in the low-frequency region, indicating the presence of a Warburg impedance. R_s_ is the internal resistance of the electrolyte, R_c_ is the charge transfer polarization resistance within the pores, CPE_c_ is the polarization capacitance of the coating [32], and CPE_dl_ is the capacitance of the double electric layer between the substrate and the coating [33]; R_ct_ denotes the charge transfer resistance between the substrate and the coating and the outside world [32], CPE_sf_ is the capacitance between the substrate and the chlorine-containing salt film, R_sf_ is the charge transfer resistance between the chloride-containing salt film inside the pitting hole and the outside world, and W is the Warburg impedance [31]. A small amount of oxide in the coating is dissolved under the action of Cl^−^, resulting in a small amount of pitting, at which point the migration of material between the coating and the corrosion solution is not negligible, and thus, there is a Warburg impedance in the circuit.

The values of each component of the corrosion equivalent electrical circuit for the three specimens are shown in Table 1. The outer polarization resistance R_c_ of the three specimens gradually increases, indicating that the outer corrosion products are gradually difficult to generate and corrosion resistance is enhanced [34]. The charge transfer resistance R_ct_ between the specimen and the outside increases successively, indicating an increase in charge transfer resistance and the enhanced corrosion resistance [35]. The charge transfer polarization resistance R_c_ within the pores, together with the charge transfer resistance R_ct_ between the substrate, the coating and the outside world, and the charge transfer resistance R_sf_ between the chlorine-containing salt film within the pitting pores and the outside world, forms the polarization resistance of the specimen (R_c_ + R_ct_ + R_sf_). The polarization resistance of 6061Al was 3.48 × 10^4^ Ω·cm^2^, that of the MAO coating was 2.68 × 10^5^ Ω·cm^2^, and that of the nano-SiO_2_/MAO composite coating was 2.92 × 10^5^ Ω·cm^2^. The addition of nano-SiO_2_ particles can improve the corrosion resistance the coating. After 7 days of corrosion, the corrosion resistance of the nano-SiO_2_/MAO composite coating was an order of magnitude higher than that of 6061Al and more than 24 KΩ·cm^2^ higher than that of the MAO coating.

By comparing the impedance values of the three specimens at experimental conditions, it can be concluded that the nano-SiO_2_/MAO composite coating has the highest value, is highly corrosion-resistant, and provides good protection to the substrate.

**Figure 9:**
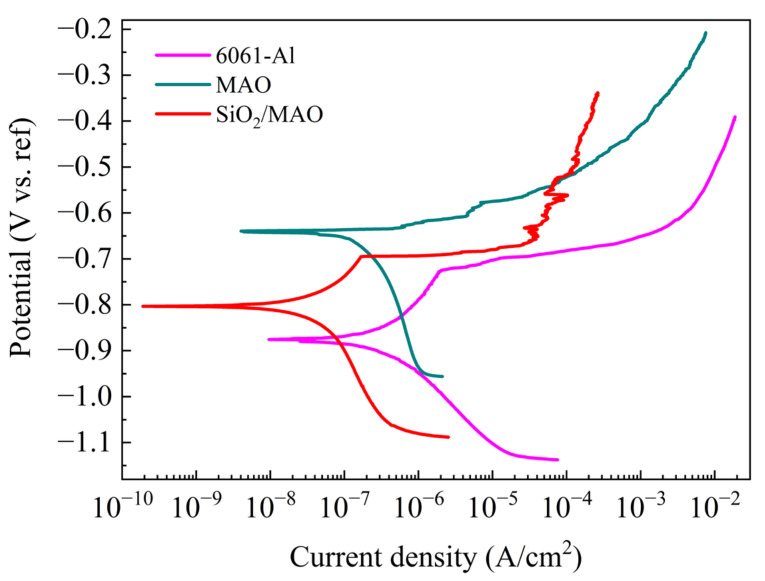
Potentiodynamic polarization curves of three specimens after immersion in 3.5% NaCl solution for 7 days.

Figure 9 shows the potentiodynamic polarization curves of the three specimens. Table 2 lists the corrosion potential values and corrosion current densities of the three specimens under corrosion for 7 days. The corrosion potential and corrosion current showed an increasing and decreasing trend, respectively. Among them, the corrosion potential and corrosion current density of the nano-SiO_2_/MAO composite coating were −0.801 V and 0.021 μA·cm^−2^, respectively. The nano-SiO_2_/MAO composite coatings have higher corrosion potentials and lower current densities, indicating that the nano-SiO_2_/MAO composite coatings can still exhibit strong corrosion resistance in polarized environments accelerated by applied currents, which is in agreement with the results of EIS. In addition, the potentiodynamic polarization curves of the 6061 Al indicated that the corrosion did not show significant passivation zones. This is because Cl^−^ in the corrosion solution destroys the passivation film on the surface of the aluminum alloy substrate [36]. According to the available experimental studies, the type of corrosion may be pitting [37], which needs to be observed in conjunction with the macroscopic morphology of the specimen.

#### 3.2.2. Corrosion Products

**Figure 10:**
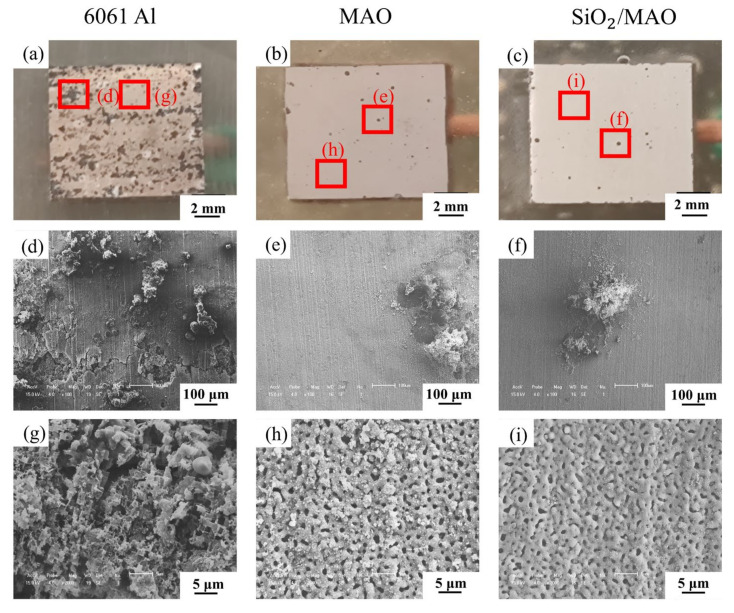
Macroscopic and microscopic appearance of three specimens after corrosion: (**a**–**c**) the macroscopic morphology of the 6061Al, MAO coating, and SiO_2_/MAO coating after corrosion; (**d**–**f**) the microscopic morphology of the corrosion pit area of the two coatings; (**g**–**i**) the microscopic morphology of the corrosion-free pit area of the two coatings.

Figure 10 shows the macroscopic and microscopic appearance of 6061Al, MAO coating and nano-SiO_2_/MAO composite coating after corrosion.

From the macroscopic point of view, the 6061Al was seriously corroded, with many corrosion pits appearing on the entire surface. Some corrosion pits interconnect to form large pits, while the surface corrosion pits of nano-SiO_2_/MAO composite coating are the least (Figure 10a–c). The magnified view of the corrosion pit locations in the three specimens (Figure 10d–f). Many corrosion pits and slight cracks were observed on the surface of the 6061Al. Meanwhile, corrosion products gathered in the corrosion pits, and some of them fell off (Figure 10d). The corrosion products accumulate on the surface of the MAO coating with obvious corrosion pits (Figure 10e). Corrosion products also accumulated on the surface of the nano-SiO_2_/MAO composite coating, but no obvious corrosion pits were observed (Figure 10f). The local magnification of the slightly corroded positions in the three specimens (Figure 10g–i). The corrosion products produced by 6061Al are loose and flocculent (Figure 10g). The micropores generated by micro-arc oxidation can be seen on the surface of both the MAO coating (Figure 10h) and the nano-SiO_2_/MAO composite coating (Figure 10i). However, there are obvious corrosion products deposited around the micropores on the surface of the MAO coating, while only trace corrosion products exist on the surface of the nano-SiO_2_/MAO composite coating.

**Figure 11:**
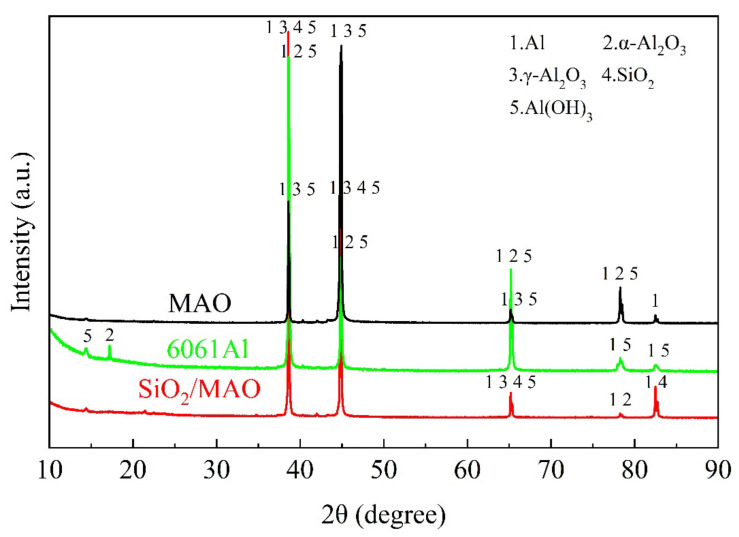
Surface phase composition of three specimens after corrosion.

Figure 11 shows the phase composition of 6061Al and the MAO coating and the nano-SiO_2_/MAO coating after corrosion. The metastable γ-Al_2_O_3_ phase in the coating reacts with Cl^−^ to form AlCl_3_ in an acidic environment, and then Al^3+^ combines with OH^−^ to form the corrosion product Al(OH)_3_ [38]. In addition to the phase of the coating itself shown in Figure 10, the corrosion product Al(OH)_3_ phase also appears.

As shown in Figure 11, the frequency of corrosion products gradually decreases after the micro-arc oxidation process, which further indicates that the number of corrosion products decreases and the corrosion resistance of the specimen increases. The frequency of the Al(OH)_3_ peak for the MAO coating decreased by one compared with that of 6061Al substrate, indicating that the corrosion was slowing down. However, the frequency of Al(OH)_3_ peak of the nano-SiO_2_/MAO composite coating with nano-particles dropped to the lowest, indicating that the addition of nano-particles can improve the corrosion resistance of the MAO coating.

### 3.3. The Results of Numerical Simulation Analysis for Long-Period Corrosion Prediction

**Figure 12:**
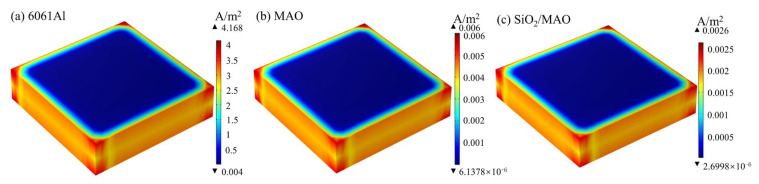
Cloud plot of corrosion current density distribution for three specimens.

The above global electrochemical experimental data were used for long-cycle corrosion prediction analysis. Figure 12 shows the corrosion current density distribution of the three specimens. The highest corrosion current density occurs at the tip of the specimens, and there are a few areas of decreasing corrosion current density around the edges of the specimens. The corrosion current density is more uniform in most areas of the specimens, with no significant changes. The results of corrosion current density distribution are consistent with the experimental results. The corrosion current density of the nano-SiO_2_/MAO composite coating was 0.0026 A/m^2^, followed by the MAO coating at 0.006 A/m^2^, and the corrosion current density of 6061Al was 4.168 A/m^2^, which was the largest among the three specimens. It can be seen that the nano-SiO_2_/MAO composite coating greatly improves the corrosion resistance of 6061Al.

**Figure 13:**
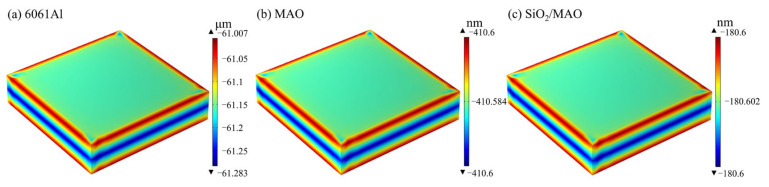
Cloud plot of the distribution of thickness reduction in specimens corroded for 10 years.

Figure 13 shows the distribution of thickness thinning over 10 years of corrosion. From the corrosion level, the corrosion amount of 6061Al is micron level, and the corrosion amount of the coating is nanometer level; therefore, the protective effect of the coating reduces the corrosion amount of 6061Al by nearly a thousand times, which effectively improves the corrosion resistance of 6061Al. The specimens of the thickness of the thinning number of large positions and the corrosion current density of large positions are consistent with the corners of the larger value, and the rest of the position is of uniform distribution.

**Figure 14:**
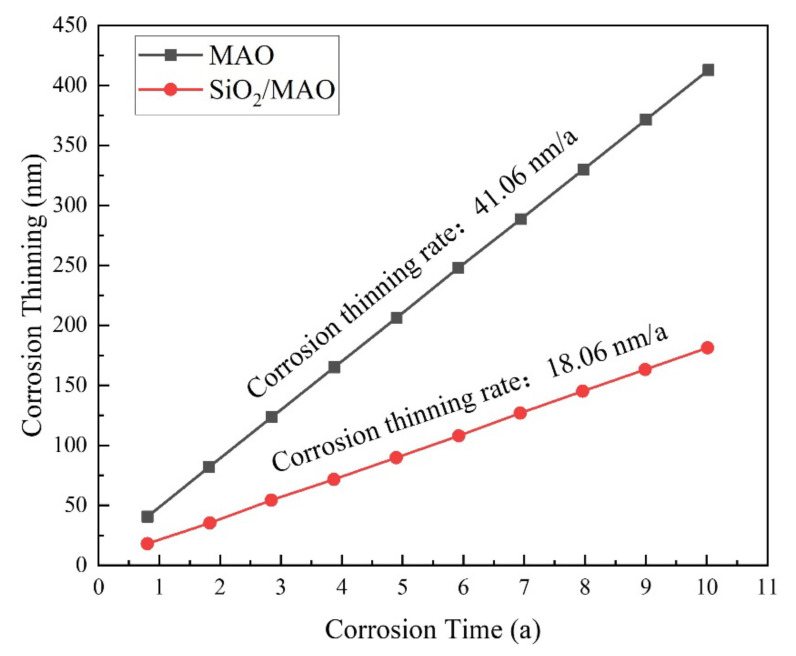
Annual corrosion thinning rate curve for coated specimens.

Figure 14 shows the annual corrosion thinning rate curve of the coating specimens. The results of the long-period corrosion prediction analysis show that the corrosion thinning rate of the MAO coating is 41.06 nm/a, the nano-SiO_2_/MAO composite coating is 18.06 nm/a, and the corrosion is slow. It meets the service life requirement of automotive battery pack surface coating.

### 3.4. Local Electrochemical Behavior

#### 3.4.1. Local Impedance Distribution

**Figure 15:**
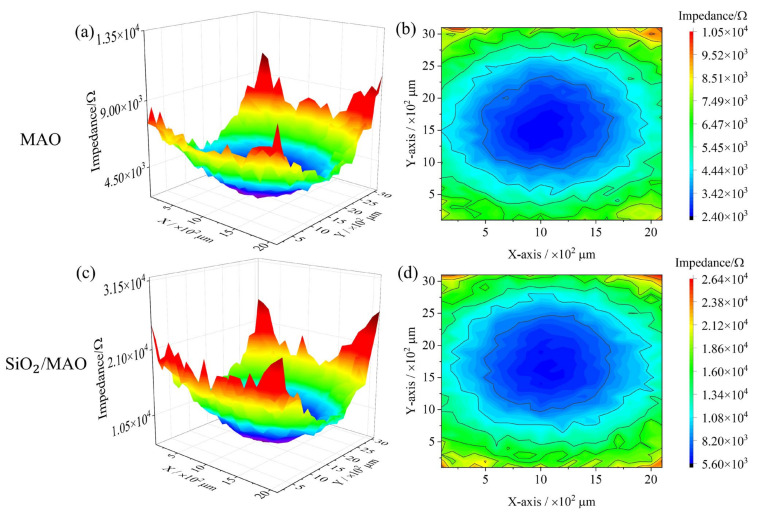
Local impedance distribution of scratched micro-arc oxidation coatings: (**a**,**b**) MAO coating; (**c**,**d**) nano-SiO_2_/MAO composite coating.

LEIS is a particularly suitable technique for testing localized corrosion such as pitting and micron level coatings. Figure 15 shows the LEIS test results for the MAO coating and the nano-SiO_2_/MAO composite coating with scratches. The scanning area is 2 × 3 mm^2^, and the damaged area is about 0.2 × 1.5 mm^2^. The scanning path is set in advance so that the damaged area is roughly in the center of the scanning area. The resistance values at the defects of the two coatings are the lowest, extending from the defects to the surrounding coatings, and the resistance values gradually increase, making the image form a valley. Under the condition of defective coating, the local impedance of MAO coating ranges from 2.40 × 10^3^ to 1.05 × 10^4^ Ω, while the local impedance of nano-SiO_2_/MAO composite coating ranges from 5.60 × 10^3^ to 2.64 × 10^4^ Ω. The nano-SiO_2_/MAO composite coating still shows high corrosion resistance in the presence of defects.

#### 3.4.2. Local Corrosion Morphology

**Figure 16:**
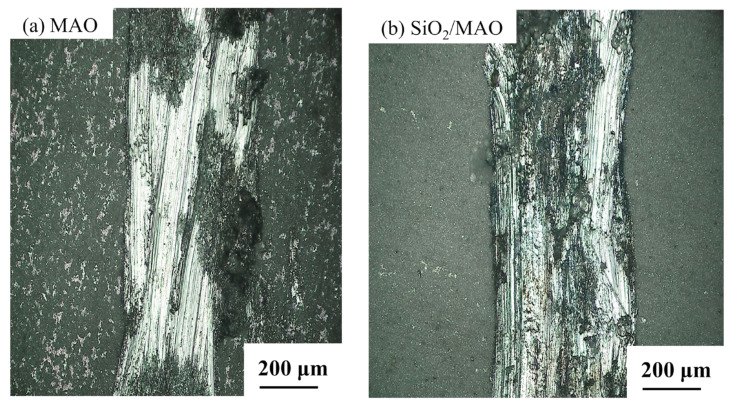
Microscopic morphology of specimens after microelectrochemical test.

Figure 16 shows the local microscopic morphology of the coating damaged after the local electrochemical test.

The obvious bright white at the scratch is the bare aluminum alloy substrate, and the black part is the location where corrosion occurs. It can be seen in Figure 16a that there are obvious large black pitting pits near the bright white area, while in Figure 16b, the damaged substrate has no obvious corrosion pits, and the overall corrosion is uniform. There are many white spots on the outside of the scratch of the MAO coating and they are uniformly distributed, indicating that the MAO coating has small local damage and the exposed 6061Al substrate can be seen. There are few white spots on the outside of the nano-SiO_2_/MAO composite coating, and the coating integrity is high. The results indicate that the nano-SiO_2_ particles can improve the corrosion resistance of the MAO coating, and can still play a good protective effect in the case of local damage of the coating, and avoid large pitting pits.

### 3.5. Corrosion Mechanism Model

**Figure 17:**
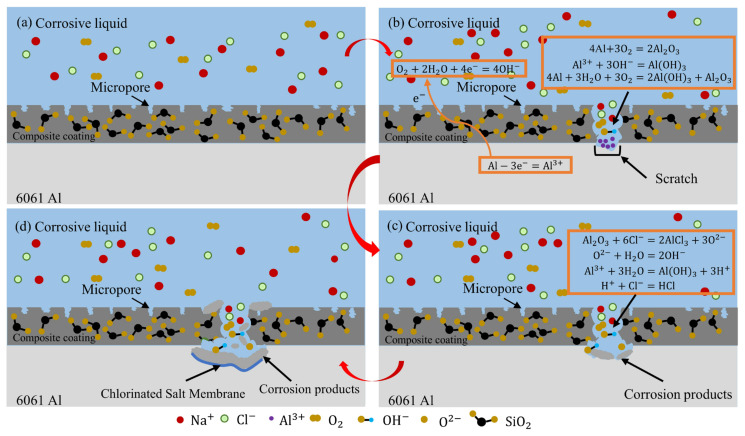
Corrosion mechanism model of nano-SiO_2_/MAO composite coating when damaged: (**a**) composite coating integrity; (**b**) initial stage of corrosion; (**c**) mid-stage corrosion; (**d**) terminal phase of corrosion.

The corrosion mechanism model of composite coating is obtained, as shown in Figure 17. The main composition of the coating is Al_2_O_3_, which is divided into α and γ crystal types. The added nano-SiO_2_ is irregularly dispersed in the coating, as shown in Figure 17a. When there is a scratch on the surface of the nano-SiO_2_/MAO composite coating, the anions in the corrosion solution penetrate into the substrate and electrochemical corrosion occurs. Meanwhile, the corrosion product Al(OH)_3_ is formed. The chemical reactions occurring during the corrosion process are described as below [14,25,31,39,40]:

Anode: Al − 3e^−^ = Al^3+^ (7)

Cathode: O_2_ + 2H_2_O + 4e^−^ = 4OH^−^ (8)

4Al + 3O_2_ = 2Al_2_O_3_ (9)

Al^3+^ + 3OH^−^ = Al(OH)_3_ (10)

4Al + 3O_2_ + 3H_2_O = 2Al(OH)_3_ + Al_2_O_3_ (11)

Al_2_O_3_ + 6Cl^−^ = 2AlCl_3_ + 3O^2−^ (12)

O^2−^ + H_2_O = 2OH^−^ (13)

Al^3+^ + 3H_2_O = Al(OH)_3_ + 3H^+^ (14)

H^+^ + Cl^−^ = HCl (15)

When the coating is partially damaged, the aluminum alloy substrate comes into direct contact with the external corrosion solution, and oxygen absorption corrosion (Equation (7)) occurs simultaneously with aluminum hydrolysis (Equation (8)). The exposed aluminum alloy forms a natural passivation film Al_2_O_3_ in the atmosphere (Equation (9)). The Al^3+^ produced by the loss of electrons of the metal aluminum combines with OH^−^ in solution to produce Al(OH)_3_ (Equation (10)), as shown in Figure 17b. Aluminum alloy reacts with water and oxygen in solution to produce Al(OH)_3_ and Al_2_O_3_ (Equation (11)). With the occurrence of corrosion, the Al^3+^ constantly generated migrated to the outside of the substrate, while Cl^−^ migrated to the inside of the substrate. The main components of the coating are α-Al_2_O_3_ and γ-Al_2_O_3_. When the coating is damaged in an acidic environment, the metastable γ-Al_2_O_3_ phase is dissolved, while the steady α-Al_2_O_3_ phase is not corroded [38]. Cl^−^ also reacted with γ-Al_2_O_3_ in the coating to form AlCl_3_ and O^2−^ at the damaged area [40] (Equation (12)). At the same time, the hydrolysis of O^2−^ produced more OH^−^ (Equation (13)), which combined with Al^3+^ to form Al(OH)_3_ and accumulated in the damaged places, as shown in Figure 17c. The Al(OH)_3_ phase was found in the coating via XRD analysis (Figure 11). Al^3+^ also reacts with water to produce Al(OH)_3_ and H^+^ (Equation (14)), which acidify the solution (Equation (15)). The diversification of corrosion behavior of 6061 Al accelerates the corrosion rate. A “small anode-big cathode” electrochemical corrosion system composed of a small exposed 6061Al substrate as the anode and a large intact nano-SiO_2_/MAO composite coating as the cathode further promotes the pitting corrosion occurrence. When the coating is damaged, the corrosive fluid rapidly corrodes the substrate along the bottom of the hole, leaving the surrounding coating in a suspended state, as shown in Figure 17d. As corrosion intensifies, the 6061 Al substrate is corroded more and more, and the suspended coating collapses inward due to its own weight, and eventually form pitting pits.

## 4. Conclusions

In this paper, three corrosion behaviors of 6061 Al substrate, MAO coating and nano-SiO_2_/MAO composite coating with nano-SiO_2_ particles were studied, and the following conclusions were drawn:

(1) The main composition of the MAO coating and nano-SiO_2_/MAO composite coating is Al_2_O_3_, and the thickness of nano-SiO_2_/MAO composite coating reaches 9.6 μm. Nano-SiO_2_/MAO composite coating has fewer micropores on the surface, indicating that nano-SiO_2_ particles have a good sealing effect.
(2) The global electrochemical test results showed that the electrochemical impedance film value and corrosion current density of nano-SiO_2_/MAO composite coating increased by 9% and decreased by 87% compared with MAO coating, respectively. The local electrochemical test results showed that the local impedance of MAO coating ranges from 2.40 × 10^3^ to 1.05 × 10^4^ Ω, while the local impedance of nano-SiO_2_/MAO composite coating ranges from 5.60 × 10^3^ to 2.64 × 10^4^ Ω, which more than doubled the local impedance in the condition of defective coating. It can be seen that nano-SiO_2_/MAO composite coating still shows high corrosion resistance in the case of defects.
(3) Based on the results of the global electrochemical experiments, the COMSOL simulation program was established to simulate the long-period corrosion experiments of the coatings and to predict the annual corrosion thinning rate of the coatings by using the Nernst–Planck equation and the Butler–Volmer equation. The numerical simulation results show that the nano-SiO_2_/MAO composite coating has the lowest corrosion current density of 0.0026 A/m^2^, the slowest corrosion thinning rate of 18.06 nm/a, and the strongest durability, which can satisfy the service life requirement of the surface coating of the battery pack.
(4) The corrosion mechanism model of nano-SiO_2_/MAO composite coating was established. When the coating is damaged, the 6061Al substrate is corroded by oxygen, and the γ phase of the composite coating reacts with Cl^−^ in the corrosion solution to finally form the corrosion product Al(OH)_3_. As the corrosion time grows, the coating collapses inward due to its own weight and eventually forms pitting pits.

## Figures and Tables

**Table 1:** Values of components in the corrosion equivalent electrical circuit for different specimens.

Specimen	R_s_ (Ω·cm^2^)	CPE_c_ (Ω^−1^·cm^−2^·s^−n^)	R_c_ (Ω·cm^2^)	CPE_dl_ (Ω^−1^·cm^−2^·s^−n^)	R_ct_ (Ω·cm^2^)	CPE_sf_ (Ω^−1^·cm^−2^·s^−n^)	R_sf_ (Ω·cm^2^)	W (Ω·cm^2^)	χ^2^
6061Al	7.31	-	-	1.08 × 10^−5^	3.48 × 10^4^	-	-	-	7.63 × 10^−4^
MAO	20.40	1.43 × 10^−7^	8.84 × 10^2^	4.71 × 10^−6^	2.63 × 10^5^	9.37 × 10^−6^	3.72 × 10^3^	7.43 × 10^3^	2.91 × 10^−4^
SiO_2_/MAO	21.58	1.71 × 10^−7^	8.66 × 10^2^	5.47 × 10^−6^	2.87 × 10^5^	1.89 × 10^−6^	3.66 × 10^3^	7.64 × 10^3^	2.95 × 10^−4^

**Table 2:** The corrosion potential E_corr_ (V) and corrosion current density I_corr_ (μA·cm^−2^) of the three test specimens.

Specimen	6061Al	MAO	SiO_2_/MAO
E_corr_	−0.860	−0.750	−0.710
I_corr_	2.020	0.758	0.504

## Data Availability

The data that support the findings of this study are available from the corresponding author upon reasonable request.
